# An evolutionary account of vigilance in grief

**DOI:** 10.1093/emph/eox018

**Published:** 2017-12-18

**Authors:** Claire White, Daniel M T Fessler

**Affiliations:** 1 Department of Religious Studies, California State University, Northridge, CA, USA; 2 Center for Behavior, Evolution, and Culture and Department of Anthropology, UCLA, Los Angeles, CA, USA

**Keywords:** grief, evolutionary psychology, vigilance, cognition, signal detection theory, corpse

## Abstract

Grief is characterized by a number of cardinal cognitive symptoms, including preoccupation with thoughts of the deceased and vigilance toward indications that the deceased is in the environment. Compared with emotional symptoms, little attention has been paid to the ultimate function of vigilance in grief. Drawing on signal-detection theory, we propose that the ultimate function of vigilance is to facilitate the reunification (where possible) with a viable relationship partner following separation. Preoccupation with thoughts about the missing person creates the cognitive conditions necessary to maintain a low baseline threshold for the detection of the agent—any information associated with the agent is highly salient, and attention is correspondingly readily deployed toward such cues. These patterns are adaptive in cases of an absent but living partner, but maladaptive in cases of the death of a partner. That they occur in the latter likely reflects the intersection of error-management considerations and the kludge-like configuration of the mind. We discuss results from two previous studies designed to test predictions concerning input conditions and individual differences based on this account, and consider the implications of these findings for mainstream bereavement theories and practices.

## INTRODUCTION 

The death of a loved one is a ubiquitous human experience. Although how grief is expressed is largely determined by societal norms, the human experience following bereavement is remarkably similar across cultures, especially for predominant emotional symptoms such as sadness [1]. Grief is also characterized by cognitive symptoms, such as preoccupation with thoughts about the deceased, vigilance toward detecting cues that the deceased is in the vicinity, and rumination over the circumstances of death [1–3]. The symptoms of grief are among the most intensely stressful experienced by humans, and, consonant with this, are likely to be detrimental to biological fitness [4–6].

From an evolutionary perspective, grief is an especially puzzling phenomenon—ceteris paribus, we should expect natural selection to minimize or eliminate responses that disrupt the individual’s ability to address current challenges and opportunities, and that entail direct somatic costs. Many investigators have sought to make sense of the symptoms of grief (most notably [7–10]). From an evolutionary perspective, these explanations largely fall into one of two categories. The first group of theories is ‘by-product’ accounts. According to one version of the by-product account, grief in the context of death is an unavoidable by-product of a more common separation response, a response that would have aided reunification (which was adaptive) in our ancestral environment, but which has always been fruitless (and thus maladaptive) in the context of death [7]. Just as some non-human animals (e.g. zebra finches [12]), vocalize their distress when separated and engage in searching behavior that adaptively aids reunification, humans engage in searching—at a variety of levels of consciousness—for a loved one when circumstances suggest that the beloved is lost. We term this version ‘the reunion account’ because in the context of separation short of death, searching is adaptive. In this account, the costs involved in grief are thought to be outweighed by the benefits conferred by separation responses. While the presence of these responses in the case of death simply reflect constraints on optimality—separation is such a powerful elicitor of the responses that it leads to distress and searching despite awareness of the impossibility of reunion. In other related theories, grief is viewed as a by-product of the emotional and cognitive systems that, respectively, motivate maintaining contact in close relationships, and generate enduring mental models of others—functions that are essential when the other party is alive, but are futile when the partner is dead [10, 13, 14].

In contrast to by-product explanations, ‘adaptation’ theories have proposed that at least some symptoms (typically rumination and sadness) in grief had direct adaptive benefits for the bereaved in ancestral environments, including helping the individual to cope with the terminal loss of a loved one by prompting the reassessment of plans, priorities, and relationships; detaching from the lost agent and engaging in new relationships; signaling a changed status to others and thus eliciting sympathy and resources; and communicating one’s suitability as a trusted relationship partner to the social group [8, 9, 15].

Despite over a century’s worth of theoretical developments and research, there is little consensus on the ultimate reasons for grief—i.e. why grief evolved—[7–10, 13, 14, 16]. Rather than attempting to adjudicate among the many competing holistic accounts of grief proposed by prior authors, we instead begin with the assumption that grief is not a singular phenomenon, and, correspondingly, is likely to have multiple consequences with regard to fitness. In short, we argue that, to understand the evolution of grief, it must first be disaggregated into meaningful subtypes based on the potential adaptive consequences of each. Furthermore, the bereaved typically evince symptoms that rise and fall as a function of time [17, 18]. A convincing evolutionary account of a given component of grief must therefore explain both the adaptive significance of the given feature and the implications of its characteristic time-course.

Consonant with the above, contemporary scholars have begun to make headway by parsing the experience of grief according to the cardinal (i.e. main diagnostic) symptoms, and investigating how each may have been shaped by natural selection. To date, foremost in the list of candidates for such treatment has been low mood. The bereaved often experience minutes-long episodes of intense sadness [19]. Many also experience depressed mood in general, a pattern that peaks in frequency between 1 and 6 months post-loss and then declines [18]. Scholars have proposed a variety of direct adaptive benefits that sadness provides for the bereaved. For instance, Nesse *et al.* have argued that sadness has multiple functions depending on context; in the context of grief, sadness is thought to foster disengagement from commitments to a deceased loved one and signal a changed status to others, thus eliciting sympathy and resources [9, 20]. Other researchers have contended that sadness functions in grief as a hard-to-fake signal (i.e. it entails significant costs that largely preclude dissimulation) that communicates the bereaved’s capacity to form strong bonds, and indexes the bereaved’s commitment to the deceased, thereby communicating to others the bereaved’s suitability as a trusted relationship partner [8, 9, 15]. Theories such as these are important in part because they overcome the main objection that mainstream bereavement researchers have toward taking evolutionary approaches seriously, i.e. that they purportedly do not generate testable predictions [16].

In contrast to the emotional components of grief, less explanatory attention has been paid to the cognitive facets of this experience, including the contents of thoughts, and changes in information processing. Existing evidence indicates that people are intensely preoccupied with unrelenting thoughts about the deceased, thoughts that are often subsumed under the categories of ‘yearning for reunion’ or ‘searching’ in traditional research. Like affective features, these cognitive symptoms have a typical time course, beginning at the onset of separation, peaking at around 1 month, and continuing to lessen until around 6–12 months, when most people return to post-bereavement functioning [21, 18]. Importantly, vigilance toward detecting cues that the deceased is in the environment increases; during bereavement, any information associated with the agent is highly salient, and attention is correspondingly readily deployed toward such cues [3, 22, 23].

In this paper, building upon our earlier work [24, 25], we take the first steps toward providing a cognitive evolutionary account of vigilance in grief by examining the adaptive significance of the predominant cognitive reactions following the onset of bereavement. This theory builds upon, and extends, the ‘reunion account’, which views of the origins of grief as a separation response aimed at promoting reunification with the lost agent. Crucially, however, we focus only on explaining the adaptive significance of vigilance in grief without assuming that the reunion account explains other symptoms—we remain uncommitted on the latter point, and, given the complexity of grief, recognize that it may not be a unitary phenomenon, and thus that other explanations may apply to other features. We focus on a number of considerations, including (i) the kludge-like configuration of the mind (i.e. the agglomeration of ad hoc solutions), such that the optimality of responses can be constrained by differential processing of information by different mental mechanisms, and (ii) adaptive responses to ambiguous versus definitive cues. This account generates testable predictions concerning input conditions and individual differences, and we will present summaries of the results of a series of studies we designed to test these [24, 25]. The theory also motivates new lines of investigation on the cognitive processes underpinning grief, and has potential implications for mainstream bereavement theories and practices.

## VIGILANCE IN GRIEF: AN EVOLUTIONARY ACCOUNT

As we have outlined, one common component of bereavement is vigilance toward detecting cues that the deceased is present in the vicinity. Information associated with the agent is highly salient, with attention being readily deployed toward such cues. These processes of attention allocation likely occur outside of conscious awareness. One indication that they are ongoing is the common experience of misperception following bereavement [26]. Particularly in the first few months following the death of a loved one, many bereaved individuals have the disquieting experience of interpreting sights and sounds as having been caused by the deceased—generally followed immediately by the realization that the given individual is dead. We term such experiences ‘false recognitions’. For example, Parkes [10] provided common examples of hearing the deceased’s footsteps on the stairs, or hearing them in the street. These (and associated) experiences have been reported across cultures, especially for children who have recently lost a parent, and adults who have recently lost a spouse [18, 22, 26–31].

There is reason to believe that these experiences are remarkably common in bereavement. For example, in reviews of studies conducted in a variety of cultures, researchers have found that around half of those surveyed have experienced false recognitions of deceased loved ones, especially deceased spouses [29, 32–35]. Investigators note that these misperceptions appear to be especially common during the first few months following bereavement, and report that they decline over time [33]. For instance, in Grimby’s [34] sample of elderly women, following the death of a spouse, 30% reported hearing the deceased at Month 1, compared with 19% at Month 2, and 6% at Month 3. Similarly, 26% reported seeing the deceased at Month 1, compared with 19% at Month 2 and 12% at Month 3 [26]. These experiences can elicit feelings of fear or reassurance, depending on the cultural context within which they occur, including cultural narratives about the deceased, an afterlife, and expectations and norms surrounding the experience of mourning [10, 34, 35].

Importantly, holding cultural meaning-making frames aside, the experience of false recognitions can be understood as underpinned by the automatic misperception of external stimuli as being caused by a particular agent. To be clear, false recognitions are not the same as hallucinations; they do not occur in the absence of external, or relevant, stimuli, and they are identified as a ‘misperception’ by the perceiver, at least inasmuch as, regardless of the cultural frame of understanding, the perceiver subsequently recognizes that the given sight or sound does not reflect the presence of the still-living agent—indeed, for perceivers who do not subscribe to ghost beliefs and the like, the sight or sound is quickly recategorized as having no connection with the agent whatsoever [36].

From a cognitive perspective, false recognitions can be understood as a product of a low baseline threshold for detecting the target person in the environment, and, relatedly, indicate that the grief process is incomplete. On our account, cognitive mechanisms responsible for quick-and-dirty interpretation of stimuli, the so-called low-road aspects of perception, continue to represent the deceased as an agent capable of producing sights and sounds that impinge on the observer. At the same time, cognitive mechanisms responsible for slower, more reflective processing, the so-called high-road components of perception, no longer represent the agent in this manner—hence the disquieting conflict between the initial interpretation of sights or sounds as caused by the loved one, followed by the subsequent recollection that the loved one is dead, and realization that the initial interpretation must be erroneous. Eventually, however, representations of the deceased become sufficiently reformulated such that their influence on relevant aspects of cognition becomes uniform—the loved one is represented as no longer a viable relationship partner at all levels of representation, with the result that sights and sounds are no longer perceived as stemming from the loved one, and false recognitions diminish and are eventually no longer experienced [3, 18, 37].

We view false recognitions as best understood within the framework of the reunion account of grief. Seen in this light, the ultimate function of preoccupation and vigilance is to facilitate reunification (where possible) with a viable relationship partner following separation. Preoccupation with thoughts about the missing person creates the cognitive conditions necessary to maintain a low baseline threshold for the detection of the agent—together, these symptoms serve to detect cues of the person’s presence in the immediate vicinity. Understood in this manner, these patterns are not a misfiring of other mechanisms, with corresponding squandering of cognitive resources, but rather the product of a correctly functioning adaptive mechanism responding to the absence of a significant other from the immediate environment [25]. Natural selection favors decision-making systems that calibrate signal detection to maximize expected value [38], a phenomenon first described in the evolutionary literature as *the smoke-detector principle* [39], and subsequently elaborated in regard to psychological mechanisms as *error-management theory* [40, 41]. Here, the costs of maintaining a given detection threshold are compared with the benefits obtained should reunion with that partner be achieved, multiplied by the probability that, at the given level of vigilance, reunification will occur. Across human evolution, when the relationship at issue was a valuable one, the potential cost of a signal-detection miss (i.e. failing to register evidence of the presence of the missing partner) will generally have been greater than the costs of a false alarm (i.e. erroneously registering spurious stimuli as indicative of the partner’s presence).

At least three important considerations factor into such decisions, considerations that loom particularly large in small groups such as those characteristic of ancestral human societies. First is the availability of suitable alternative partners—which, necessarily, would have been constrained by group size. Second is the potentially sizeable transaction costs involved in seeking out a new relationship partner. Third, temporary separation—which can have many causes—is generally more common than permanent separation by death. Thus, especially in the early phases following separation (when reunification is more likely), the costs of erroneously prematurely abandoning efforts at reunification and pursuing replacement relationships will usually have exceeded the costs of erroneously persisting in efforts aimed at reunification. Consequentially, bereaved individuals sustain a low baseline threshold for the detection of the missing agent. Of course, death is a permanent separation, and, in such instances, a correctly functioning system should eventually alter representations of the agent such that the deceased is no longer viewed as a potential relationship partner. *Ceteris paribus*, the probability that reunification will occur should decline as a function of the passage of time. Correspondingly, preoccupation symptoms dampen with time since the onset of bereavement [18, 21].

Intrinsic to the above logic, in addition to the passage of time, the fitness value of the relationship partner will affect the calibration of detection for the missing loved-one. Not all relationships are equal, and therefore, not all efforts to recover a relationship will be equal. For example, the losses of close genetic relatives, and of individuals having high reproductive value (i.e. adolescent children) have been found to be the most painful [42–44]. Another factor that should determine cognitive symptoms following bereavement is the amount of investment that has gone into the relationship (e.g. time, resources). High investment indicates that the costs of replacing the given relationship partner will be similarly high, and hence the fitness consequences of the loss are great. Clinical research reveals that the level of attachment to the deceased—a subjective proxy for relationship investment—is a good predictor of overall grief intensity [3, 22, 23], and, according to the account proposed here, of that cognitive component of grief consisting of increased vigilance.

## PREDICTIONS AND RESEARCH

Our evolutionary theory of vigilance in grief generates several key predictions about the input conditions that will affect the cognitive symptoms of grief following the onset of bereavement. As outlined, and commensurate with mainstream bereavement research, cognitive symptoms of grief should diminish with time since onset of bereavement, and should be heightened with increasing levels of attachment to the deceased. Crucially, the theory also generates novel predictions. These involve the effect on cognitive components of grief following, respectively, indications that the agent is in the vicinity, alive, and a viable relationship partner, or, conversely, indications that the agent is dead. We recently tested these predictions in two cross-sectional studies [24, 25]; we outline them in more detail below.

### Study 1: input that the agent is alive

One key prediction arising from this evolutionary account of vigilance in grief is that input suggesting that the deceased is alive will exacerbate the cognitive symptoms of grief, including the experience of false recognitions—the more indications there are that reunification with the missing partner is possible, the more that the threshold for detecting the presence of the partner should be lowered, and the longer this condition should persist. Because realistic photographs and other media were not components of the stimulus environment in which the mechanisms underlying grief evolved, they may be processed at least to some degree as veridical. It therefore follows that exposure to such images will positively correlate with the experience of false recognitions. Just as in the case of images of watching eyes [45, 46], the functioning of relevant mental mechanisms will be affected by these evolutionarily novel stimuli despite propositional knowledge that they are inanimate, producing input that interferes with the process of forming new cognitive representations of the deceased. Indeed, neurobiological research reveals that viewing photographs of familiar others activates facial recognition systems (i.e. personal identification networks) as though the individuals viewed were physically present [47, 48]. Thus, frequently viewing realistic photographs of the deceased is likely to be a perpetuating factor in the experience of false recognitions, and hence this behavior will positively correlate with such experiences.

White and Fessler [25] tested this prediction in a survey of 164 recently bereaved (<25 months) individuals in the USA and the UK. Given the obstacles to studying grief experiences following the loss of a human partner, the study examined respondents’ experiences following the death of a beloved dog or cat. Prior research indicates that such bereavement is similar to that obtaining in human relationships, yet the death of a pet lacks cultural practices and other social mores that can influence the expression of grief and related phenomena [49–51].

White and Fessler examined the extent to which variance in false recognitions was predicted by (i) frequency of viewing images, (ii) elapsed time since pet’s death and (iii) the level of attachment to pet. Multiple regression was used to analyze the contribution of each of the three variables to the extent of false recognitions, and to weigh the accuracy with which the frequency of viewing images predicted false recognition scores relative to the contributions of attachment and elapsed time. Frequency of viewing images of the pet, attachment to the deceased, and time elapsed since pet’s death all correlated significantly with the extent of false recognitions experienced by the bereaved. Despite the complexity of the relationships between the variables examined, the strongest predictor of false recognitions was the frequency of viewing photographs of the deceased. Viewing behavior made the largest unique contribution, such that level of attachment and time elapsed since death could be removed and this factor would still accurately independently predict scores on the false recognition questionnaire. Although correlation is not causation, these findings suggest that, while attachment may partly drive the frequency of viewing images, it is that frequency that affects the extent of false recognitions, not attachment alone. The strongest predictor, making a unique contribution, was the frequency of viewing photographs of the deceased. This pattern is consonant with the premise that, being evolutionarily novel, realistic photographs are treated as reliable cues that the agent remains a viable relationship partner.

### Study 2: input that the agent is dead

Increased vigilance for an absent relationship partner is dysfunctional when death, not mere separation, is the cause of the partner’s absence. Reliable cues of death should therefore diminish vigilance and raise detection thresholds. Distinguishing between living and dead agents, be they human or animal, is a critical and evolutionarily ancient adaptive challenge. As we have discussed, the baseline threshold for detecting signs indicating that a target individual is alive is especially low for recently bereaved individuals. Regarding issues of signal detection, one of the most informative stimuli should be the immediate presence of a physical body. However, this is not as straightforward a cognitive mapping task as it might seem.

Adopting a signal-detection perspective, Barrett and Behne [52] cogently argue that a basic asymmetry characterizes the task of differentiating living agents from dead entities, as, for example, erroneously concluding that an animal is dead when, in fact, it is merely not moving will generally be costlier than erroneously assuming that an animal is merely not moving when it is, in fact, dead. Correspondingly, Barrett and Behne propose that the influence of cues of death on the low-level recategorization of the agent from living to dead should be fundamentally contingent on their reliability. Cues such as grievous injuries and extensive disruptions to the body envelope have reliably indexed death throughout our species’ evolutionary history. Thus, evolved mechanisms that distinguish between living agents and dead entities should be highly responsive to such indices, with corresponding implications for subsequent vigilance. In contrast, visual exposure to an intact corpse provides ambiguous cues regarding death, leading to a state of ambivalence as to the status of the individual (see also [53]). In the context of bereavement, considered with regard to high-road information processing (in which propositional knowledge creates a rich context for interpretation and emotional response), seeing the wholly intact but immobile body of the deceased can be a deeply disturbing experience, one that cements for the bereaved the reality of the loss. However, considered with regard to low-road information processing (in which propositional knowledge may play little or no role, and responses are more strongly driven by the cue structure of the stimulus), an intact but immobile body constitutes an ambiguous stimulus, as this is not a reliable cue of death. Convergent evidence of the functional importance of these considerations comes from observations of wild chimpanzees’ behaviors that are analogous to those we would expect humans to exhibit when uncertain whether a person is dead: chimpanzees inspect and manipulate the newly deceased individual’s body, touching its face, lifting the body off the ground, and probing it. Later, conspecific investigation turns to shaking, dragging, and even beating the corpse [54, 55].

In modern Western cultures, treatment of the corpse may greatly exacerbate cue ambiguity, as the corpse is often processed to appear as lifelike as possible; correspondingly, it is common for the bereaved to express ambivalence and even apparent disbelief upon seeing the body, captured by statements such as ‘I can’t believe she’s dead’ or ‘he looks so peaceful, like he’s sleeping’. We proposed that, because of signal-detection considerations wherein the costs of erroneously presuming death will generally have been higher than the costs of erroneously presuming life, seeing an intact corpse will often be insufficient to diminish vigilance for that agent that occurs via low-road cognitive pathways. In other words, when it comes to viewing the deceased, it is not whether one sees the corpse, but rather the state of that corpse, that matters [24].

We surveyed 142 bereaved pet owners in the USA and the UK who had recently (<12 months) experienced the death of a beloved dog or cat. Replicating our earlier results [25], we found a significant positive correlation between the level of attachment and the extent of false recognitions, and a significant negative correlation between time elapsed since the pet’s death and the extent of false recognitions. Examining our thesis with regard to corpse exposure, as predicted, we found no significant impact of exposure to an intact corpse on false-recognition experiences, but a significantly negative correlation between exposure to a non-intact corpse and the frequency of false recognitions. Again, multiple regression was used to analyze the contribution of each of the variables to the extent of false recognitions. Seeing a non-intact corpse was the strongest predictor (in a negative direction) of the extent of false recognitions. This suggests that seeing the intact corpse of a loved one is not sufficient to alter the low baseline threshold for detecting cues of the missing agent in the environment; in contrast, by providing reliable indications that the agent is dead, exposure to a non-intact corpse raises the detection threshold for such cues, thus lowering vigilance for signs of the lost agent.

## IMPLICATIONS

Our research to date has focused on specific features of changes in information processing during bereavement. However, this work has limitations, much remains to be done, and caution is needed in extrapolating from the results of our research to other aspects of grief. First, our work to date is cross-sectional, and thus involves inferring causal relationships from correlations. As noted above, although the patterning of our results is consonant with our interpretations, we cannot rule out alternative causal pathways. Second, our studies deal with the loss of a pet, not human loss. While similar, the two processes are not identical. For instance, human partners provide additional resources (e.g. reproduction, care-taking) that pets cannot. Third, it is important to reconcile our findings with potentially contrasting results reported elsewhere in the literature. For example, we found that bereaved individuals who were exposed to a non-intact corpse (some of whom witnessed their pet’s traumatic death) reported fewer false recognitions than those who were exposed to an intact corpse (none of whom witnessed their pet’s traumatic death). However, other research on bereavement shows an association between the beloved’s having suffered a traumatic death and poorer health outcomes for the bereaved, including the extent to which the bereaved experience intrusive and distressing thoughts about the event [56–58]. At present, these reports do not allow us to determine whether the key factor here is (i) that the bereaved possesses propositional knowledge that the deceased died in a traumatic manner (in which case the bereaved might suffer greater distress and intrusive ideation either due to the fruitless application of a normally adaptive empathy response, or due to the adaptive application of mechanisms that assess hazards that may confront the bereaved directly), or (ii) exposure to a traumatized corpse, and crucially, the relationship between intrusive thoughts about the deceased and false recognitions. To adjudicate among these possibilities, future research on the effects of having lost a loved one to a traumatic death must investigate more closely the informational environment presented to the bereaved.

Our account generates a variety of tractable avenues for exploring the origins and functions of symptoms underpinning the cognitive components of grief. For instance, one clear prediction is that false recognitions should be present when a loved-one is absent, such as a child leaving for college or a spouse on military duty, especially when the duration of absence (and thus, return) is uncertain. While there is anecdotal evidence for such a pattern, there is a dearth of empirical research. Likewise, we predict heightened false recognitions when the absence is unexpected. In the environment of evolutionary adaptedness, valued partners will often have been away (hunting, visiting kin in other bands, etc.), hence there is every reason to expect that the mind can represent the separation as temporary, with an expected end point—in which case there is no problem to be solved, and thus no grief needed. The more uncertainty there is regarding the possible return (e.g. military deployment versus vacation), the more we might expect cognitive grief-like symptoms, including false recognitions, to appear. Furthermore, technologically mediated contact (phone calls, emails, texts, videochats, etc.) likely suffices to greatly reduce the experience that the beloved is absent, hence even military deployments may not elicit grief-like symptoms in the contemporary West; the impact of such technology could be tested by, for example, comparing the experiences of spouses of sailors deployed aboard nuclear submarines—who are often unable to contact home for long periods—with the experiences of spouses of sailors deployed aboard surface vessels.

Another prediction clearly following from our research, and from Rusbult’s [59] investment model of relationships, is that the extent to which one experiences heightened vigilance (and false recognitions) should be inversely related to the number of available alternative relationship partners. Relatedly, although some existing research indicates that losing a loved one to trauma exacerbates bereavement over and above any contribution of the suddenness of the death [56–58], our perspective predicts that suddenness should indeed be an exacerbating factor, hence this association merits additional investigation. Witnessing the progressive decline of a loved one prior to their death due to senescence or illness is common in contemporary Western societies, and would not have been uncommon in ancestral conditions. Such a process allows the survivors to recategorize the beloved as increasingly less agentic, and thus to gradually discount the value of the given relationship; we should therefore expect that, all else being equal, death proceeded by progressive decline should elicit less bereavement-linked vigilance than should sudden and unexpected death, and may often be accompanied with a sense of relief [60]; in ancestral societies, and also in contemporary economically marginal non-industrial societies, progressive decline would be expected to sometimes even lead to active geronticide or abandonment [61, 62].

As an aside, we also note here that our position predicts that, if progressive decline includes punctuated decrements (e.g. a debilitating stroke, followed by prolonged impairment leading to death), bereavement may occur prior to the death of the beloved (e.g. caregiver grief in end-stage dementia [63, 64]). In this case, vigilance takes the form of attending to indications that the sudden decrement has been reversed, and thus that the relationship partner has regained their prior value. Specifically, we can expect false recognitions concerning actions or utterances attributed to the patient, followed by the dispiriting realization that the patient is incapable in this regard. Here, the physical presence of the living patient is akin to the presence of an intact corpse, in that error-management considerations dictate that the bereaved anticipate reunion with an intact relationship partner. Likewise, here too elapsed time should be a factor, as the expected rewards for maintaining vigilance necessary decline as increasing duration dictates decreasing probability of ‘reunification’.

In exploring many of the above issues, it may be useful to compare the consequences of the death of a valued relationship partner with those of the severance of the relationship. A small body of existing research investigates grief following divorce, with some intriguing parallels being reported. For example, in both circumstances, lack of forewarning exacerbates grief, as does dependency on the other party [65]. As discussed above, both patterns are clearly predicted by our account; paralleling our work on pet death, it would thus be instructive to examine the determinants of the frequency of false recognitions in divorce.

Although preliminary, our research suggests that it may be important to weigh carefully several considerations in the event of the death of a loved one. First, our findings suggest that more research be conducted on the effects of frequently revisiting mementos and viewing photographs of the deceased, now afforded by social media that includes memorial sites, such as Facebook pages. These behaviors may indeed provide immediate reward, as neurobiological research suggests that this may activate dopamine release and therefore temporarily enhance feelings of well-being [19]. However, our research suggests that such actions may delay the transition from cognitive preoccupation with the deceased to the dampening of such symptoms. More than a century ago, Freud [22] fiercely argued for the temporary removal of reminders of the deceased in order to allow the bereaved to successfully detach. While this has gone out of fashion, the cognitive evolutionary model proposed here suggests that, at the least, more research is needed on the consequences of these divergent practices. This is ever more important given some clinicians’ recommendations for the bereaved to contemplate mementos (especially photographs) of the deceased [66]. This recommendation is promulgated despite equivocal evidence that such actions have a positive effect on overall grief outcomes [67, 68].

Second, the medicalization of the dying process in industrialized societies and the parallel professionalization of mortuary services have radically altered the experience of having a loved one die, to the point that these nations have become outliers on the spectrum of the world’s cultures [69]. Today, bereaved individuals in industrialized societies rarely participate in the preparation of the corpse, and, indeed, have minimal exposure to cues of death of any sort; when visual contact does occur, it most often follows complex professional preparation of the corpse aimed at minimizing cues of death. Our findings underscore the need for clinically relevant investigations of the effects of viewing the corpse on the bereaved, an area that has lacked systematic enquiry in mainstream bereavement research [70]. Our research suggests that contemporary evolutionarily novel practices may be retarding at least one part of the grief process by failing to deactivate agency detection.

Despite over a century’s worth of empirical research, little is known about the conditions that alleviate or exasperate aspects of grief. What, in fact, are the consequences for the bereaved of seeing and touching the corpse? Is the experience of grief different when the bereaved is cremated or buried? In a closed casket or an open one? Is revisiting reminders of the deceased helpful or harmful to resolving the grief process? What are the consequences for the bereaved of experiencing bouts of extreme sadness? What are the consequences of freely expressing such sadness? These questions remain largely unanswered in part because mainstream bereavement research lacks a core theoretical framework within which to create, situate, and evaluate empirical research [13]. We believe that evolutionary theory can provide the foundation for such a theoretical framework, and that progress can be made by decomposing grief, a multifaceted phenomenon, into its constituent cognitive and affective components.

**Conflict of interest:** None declared. 
